# Whole Exome Sequencing Identifies Mutations in Usher Syndrome Genes in Profoundly Deaf Tunisian Patients

**DOI:** 10.1371/journal.pone.0120584

**Published:** 2015-03-23

**Authors:** Zied Riahi, Crystel Bonnet, Rim Zainine, Saida Lahbib, Yosra Bouyacoub, Rym Bechraoui, Jihène Marrakchi, Jean-Pierre Hardelin, Malek Louha, Leila Largueche, Salim Ben Yahia, Moncef Kheirallah, Leila Elmatri, Ghazi Besbes, Sonia Abdelhak, Christine Petit

**Affiliations:** 1 Institut Pasteur de Tunis, LR11IPT05, Biomedical Genomics and Oncogenetics Laboratory, 1002, Tunis, Tunisia; 2 Faculté des Sciences de Tunis, Université de Tunis El Manar, 2092 El Manar I Tunis, Tunis, Tunisia; 3 Institut de la Vision, INSERM UMRS 1120, UPMC- Paris 6, Paris, France; 4 La Rabta Hospital, Otorhinolaryngology Diseases, Tunis, Tunisia; 5 Université de Monastir, Institut Supérieur de Biotechnologie, Monastir, Tunisia; 6 Institut Pasteur, Unité de Génétique et Physiologie de l’Audition, Paris, France; 7 Laboratoire de Biochimie et de Biologie Moléculaire, Hôpital Armand Trousseau, APHP, Paris, France; 8 Department B of Ophthalmology, Hedi Rais Institute of Ophthalmology, Tunis, Tunisia; 9 Universite de Monastir, Faculty of Medicine, Fattouma Bourguiba University Hospital, Department of Ophthalmology, 5000, Monastir, Tunisia; 10 Collège de France, Paris, France; Justus-Liebig-University Giessen, GERMANY

## Abstract

Usher syndrome (USH) is an autosomal recessive disorder characterized by combined deafness-blindness. It accounts for about 50% of all hereditary deafness blindness cases. Three clinical subtypes (USH1, USH2, and USH3) are described, of which USH1 is the most severe form, characterized by congenital profound deafness, constant vestibular dysfunction, and a prepubertal onset of retinitis pigmentosa. We performed whole exome sequencing in four unrelated Tunisian patients affected by apparently isolated, congenital profound deafness, with reportedly normal ocular fundus examination. Four biallelic mutations were identified in two USH1 genes: a splice acceptor site mutation, c.2283-1G>T, and a novel missense mutation, c.5434G>A (p.Glu1812Lys), in *MYO7A*, and two previously unreported mutations in *USH1G*, *i*.*e*. a frameshift mutation, c.1195_1196delAG (p.Leu399Alafs*24), and a nonsense mutation, c.52A>T (p.Lys18*). Another ophthalmological examination including optical coherence tomography actually showed the presence of retinitis pigmentosa in all the patients. Our findings provide evidence that USH is under-diagnosed in Tunisian deaf patients. Yet, early diagnosis of USH is of utmost importance because these patients should undergo cochlear implant surgery in early childhood, in anticipation of the visual loss.

## Introduction

Deafness is the most common birth defect and the most frequent sensorineural disorder [1]. It affects 1.9 per 1000 infants at birth, and its prevalence is 2.7 per 1000 in four year old children [2]. More than 50% of congenital deafness cases have a genetic cause, of which about one third corresponds to syndromic deafness [3]. More than 400 syndromes including deafness have been described [4]. Usher syndrome (USH) is an autosomal recessive disorder characterized by combined deafness-blindness, and accounts for approximately 50% of all hereditary deafness-blindness cases [5]. The prevalence of USH is estimated at 3 to 6/100,000 [6]. Three clinical subtypes (USH1, USH2, and USH3) are distinguished according to the severity and progression of deafness, presence or absence of vestibular dysfunction, and age at onset of the visual loss due to retinitis pigmentosa (RP) [7]. USH1 is the most severe form, characterized by congenital profound deafness, constant vestibular dysfunction, and a pre-pubertal onset of RP. Six USH1 genes have been identified: *MYO7A*, *CDH23*, *PCDH15*, *USH1C*, *USH1G*, and *CIB2*, encoding myosin VIIA, cadherin-23, protocadherin-15, harmonin, sans, and calcium- and integrin-binding protein 2, respectively [8,9]. Since next generation sequencing techniques currently allow rapid and cost-effective identification of the causative mutations in deaf patients [10,11], we carried out whole exome sequencing (WES) analysis in four unrelated Tunisian patients affected by apparently isolated, congenital profound deafness, who did not carry a bi-allelic mutation in the most commonly involved gene *GJB2*. Much to our surprise, we thereby identified bi-allelic mutations in USH1 genes in the four patients.

## Patients and Methods

### Patients

**Fig 1:**
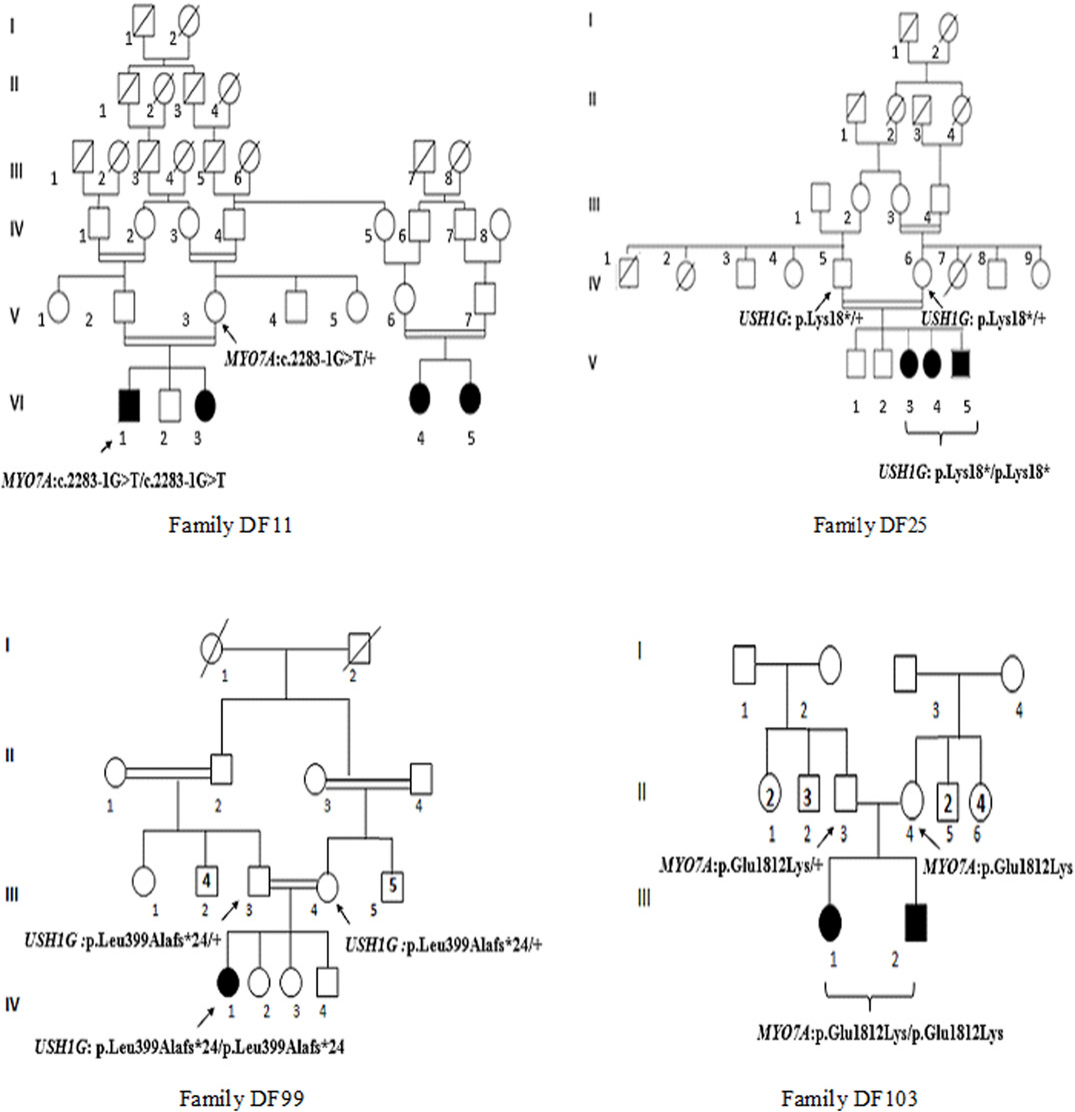
Pedigrees of the four Tunisian patients analyzed by whole exome sequencing. Squares and circles denote males and females, respectively. Filled symbols indicate deaf individuals. Arrows indicate the individuals analyzed by WES.

Four unrelated Tunisian families including one or several deaf individuals (DF11, DF25, DF99, and DF103) were studied (Fig. 1). The patients were referred from the Otorhinolaryngology Department at La Rabta University hospital in Tunis. They underwent the following clinical investigations and audiological evaluation: tympanometry, auditory brainstem response, computed tomography of the temporal bones, magnetic resonance imaging of the inner ear, ocular fundus examination, cardiac and renal ultrasonography. All the patients had bilateral profound deafness. Clinical examination and familial information were unremarkable, and did not reveal symptoms or malformations that would suggest a syndromic form of deafness. Written informed consent was obtained from the parents of all participants to the study.

### Ethics statement

This study has obtained the ethics approval (IPT/ LR11–05/Etude/05/2013) from the institutional review board of Pasteur Institute (Tunis- Tunisia- Registration number IRB00005445, FWA00010074). This study was conducted according to the principles of the declaration of Helsinki. Patients were anonymized and the corresponding code was conserved in a confidential file.

### Prescreening of *GJB2* mutations and whole exome sequencing

Genomic DNA was extracted from peripheral blood samples using the standard salting-out method [12]. First, the entire coding region of *GJB2* and flanking splice acceptor site was analyzed by Sanger sequencing in one patient of each family, i.e. DF11-VI.1, DF25-V.3, DF99-IV.1, and DF103-III.2, aged 6, 20 (with severe intellectual deficiency), 4 and 1 years, respectively [13]. These patients then underwent WES on Illumina HiSeq2000 plattform (IntegraGen: Evry, France) using Agilent Sure Select Human All Exon v2 enrichment kit. Image analysis and base calling were performed using the Illumina Real-Time Analysis Pipeline version 1.14 with default parameters. Bioinformatic analysis was based on the Illumina pipeline (CASAVA 1.8) which aligns reads to the human reference genome (hg19) with the alignment algorithm ELANDv2 (it performs multispeed and gapped alignments). Genetic variation was annotated with the IntegraGen in-house pipeline [10,14]. We achieved an average percentage of 95% of covered CCDS (Consensus Coding DNA Sequence) at 10X and 90% at 25X.The mutations identified in *MYO7A* and *USH1G* were confirmed by Sanger sequencing of the corresponding exons, using primers designed with Primer3 (http://primer3.ut.ee/) (sequences are available on request).

## Results and Discussion

According to familial information, all the patients started to walk later than normal, at about two years of age, and had balance problems, indicating vestibular dysfunction. Because of the association of profound congenital deafness and vestibular dysfunction, suggesting USH, ocular fundus examination was carried out in the patients, but abnormal findings were not reported.

Screening for mutations in *GJB2*, the gene most frequently involved in autosomal recessive deafness in Tunisia [13], by Sanger sequencing, showed that patient DF99-IV.1 carried the c.35delG mutation in the heterozygous state, whereas the other three patients were homozygous for the normal allele. WES was therefore carried out in the four patients. Based on familial history and parental consanguinity, we hypothesized that the causal mutations would be present at the homozygous state at least in three patients, and possibly at the compound heterozygous state in patient DF103-III.2, who was born to presumably unrelated parents. In the bioinformatic analysis of the results, we first excluded all the sequence variants reported in dBSNP132, 1000 genomes, Hapmap, and Exome Variant Server databases. In the second step, we focused on variants present in the coding exons and flanking splice sites. From the SNV and indel files, we selected nonsense, frame-shifting (indels), missense, and splice site mutations, as they were more likely to be pathogenic. Only the variants with a read depth greater than 5 were retained. An average of 71967 SNV and 5544 indels were found for each patient. After the filtering steps, these variants were reduced to 4 SNVs and 0 indel for patient DF25-V.3, 7 SNVs and 2 indels for patient DF11-VI.1, 12 SNVs and 2 indels for DF99-IV.1 and 2 SNVs and 1 indel for patient DF103-III.2 (S1 Table). No mutations were identified in the compound heterozygous state in any of these patients.

Much to our surprise, the bi-allelic mutations predicted to be pathogenic were all located in two USH1 genes. In patient DF11-VI.1, a splice site mutation (c.2283–1G>T) was identified in *MYO7A* (NM_001127180). This mutation has previously been reported in USH1 patients from Algeria, Morocco and France [15], but not from Tunisia. According to Alamut 2.3 software (http://www.interactive-biosoftware.com), it is predicted to abolish the splice acceptor site of intron 19 and to create a cryptic acceptor site 1 bp upstream of the original site, which could result in skipping of exon 20 in the mature transcript. In patient DF103-III.2, a previously unreported missense mutation, c.5434G>A (p.Glu1812Lys), was identified in exon 39 of the same gene (*MYO7A*: NM_000260). This mutation is predicted to have a deleterious effect on the protein by SIFT (http://sift.jcvi.org/) and Mutation Taster (http://www.mutationtaster.org/). In patients DF25-V.3 and DF99-IV.1, a nonsense mutation, c.52A>T (p.Lys18*), and a frameshift mutation, c.1195_1196delAG (p.Leu399Alafs*24), were identified in exon 1 and 2 of *USH1G* (NM_173477), respectively. According to Alamut 2.3, these truncating mutations are expected to result in the absence of synthesized protein due to mRNA nonsense mediated decay. None of the pathogenic identified mutations were described in ExAC project (last update 29 October 2014) (http://exac.broadinstitute.org/)

The presence of the mutations in the heterozygous state was shown by Sanger sequencing in all the clinically unaffected parents, except the father of patient DF11-VI.1, whose DNA was not available. We were also able to confirm the presence of the mutations, in the homozygous state, in the affected siblings of patients DF25-V.3 and DF103-III.2.

The four studied cases were a part of a group of 10 deaf families negative for *GJB2* mutations with a positive history of hearing loss which underwent WES: eight biallelic mutations were identified in 8 families (80% of resolved cases); four of them correspond to new mutations in genes responsible for isolated deafness [10], four had mutations in genes involved in Usher syndrome (this study) and two families were not resolved.

**Fig 2:**
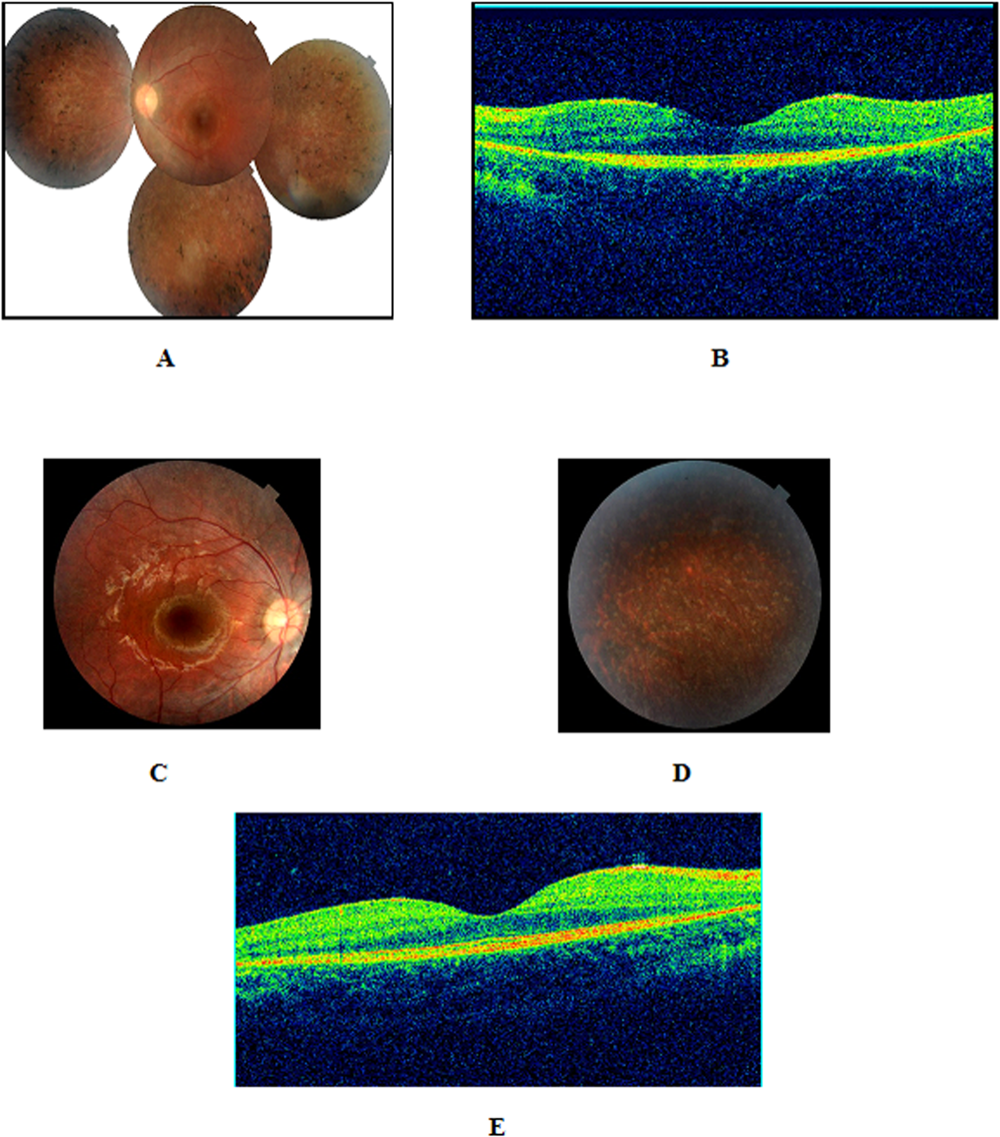
Retinal phenotypes of patients with mutations in Usher genes. **A**, Composite color fundus photograph of the left eye of a four-year-old girl (DF103-III-2) showing diffuse narrowing of the retinal arteries and hyperpigmentation in a bone-spicule configuration in the midperipheral retina. **B**, B-scan OCT imaging of the same eye showing a mild foveal atrophy (central macular thickness = 160 micrometers). **C**, Color fundus photograph of the posterior pole of the right eye of a six-year-old boy (DF103-III-1) with early stage retinitis pigmentosa shows no obvious abnormalities which may explain the misdiagnosis of the disease in some cases. **D**, Color fundus photograph of the peripheral retina showing a “salt and pepper" appearance without the classical bone-spicule pigmentation. **E**, The fovea has a normal thickness on optical coherence tomography (180 micrometers).

Because mutations in *MYO7A* and *USH1G* are associated with USH1, the patients underwent another ophthalmological examination, including optical coherence tomography (OCT) imaging (Fig. 2), and bilateral peripheral RP was diagnosed in all the patients examined, i.e., DF11-VI.1, DF11-VI.3, DF25-V.3, DF25-V.4, DF25-V.5, DF99-IV.1, DF103-III.1, and DF103-III.2.

The fact that previous routine ocular fundus examination did not detect the abnormal retinal pigmentation in any of the affected children is likely due to the presence of minor undetectable anomalies at the time when these children were first examined or to the fact that ophthalmologists are not familiar with peripheral alterations of the retina as they usually focus on the central retina and to the difficulty of making a good fundus examination in children because of absent cooperativity. Other efficient ophthalmological tests such as electroretinography and OCT imaging are needed for USH diagnosis in early childhood, but the availability of the corresponding equipment is generally limited to tertiary health centers. However, it is of utmost importance to raise awareness about the possibility that congenital deafness reveals USH, because early diagnosis of this dual sensory disorder is crucial and urges on cochlear implant in the affected children. In this respect, combining advanced ophthalmological evaluation, including funduscopy with careful examination of the peripheral retina and electroretinogram in selected cases, together with molecular diagnosis by targeted USH exome sequencing [16] or WES (this study) could be a powerful strategy.

## Supporting Information

S1 Table List of the bi-allelic mutations retained from the whole exome sequences after the filtering steps. (PDF)
