# Supplementary material for: Whole Exome Sequencing Identifies Mutations in Usher Syndrome Genes in Profoundly Deaf Tunisian Patients
Source: PLoS One. 2015 Mar 23;10(3):e0120584. doi: 10.1371/journal.pone.0120584 (PMC4370767; doi:10.1371/journal.pone.0120584)
Supplement: S1 Table — (PDF) [file pone.0120584.s001.pdf]

Table S1:

Family DF11

Gene	Refseq	Mutation	Number of reads
MACF1	NM_012090	p. Cys666Tyr	186
PPFIA4	NM_015053	p. Pro418His	32
DNAJB13	NM_153614	p.Thr285Ile	22
MYO7A	**NM_001127180**	**c.2283-1G>T**	**109**
KRTAP17-1	NM_031964	p.Gly45Ala	20
SOGA2	NM_015210	p.Arg1075Cys	47
TGFBR3L	NM_001195259	p.Pro1055Ser	16
PCGF6	NM_001011663	p.Pro27_Pro28dup	7
HOMEZ	NM_020834	p.Glu537del	22

Family DF25

Gene	Refseq	Mutation	Number of reads
RNASEK	NM_001004333	p.Pro14Ser	58
USH1G	**NM_173477**	**p.Lys18***	**23**
ZSCAN12	NM_001163391	p.Gly531Trp	71
MUC22	NM_001198815	p.Thr808Ala	199

Family DF99

Gene	Refseq	Mutation	Number of reads
OR2T35	NM_001001827	p.Leu107Arg	24
KRT18	NM_199187	p.Asp96His	28
STAC3	NM_145064	p.Ile364Phe	37
DNAH17	NM_173628	p.His776Gln	136
CEP89	NM_032816	p.Ala509Thr	84
ZNF793	NM_001013659	p.His148Asn	194
LGALS4	NM_006149	p.Pro190Leu	57
CEACAM20	NM_001102598	p.Ala198Thr	131
LHB	NM_000894	p.Met3Arg	42
LILRB1	NM_001081639	p.Asp260Glu	40
METAP1D	NM_199227	c.541-1G>A	175
TMEM2	NM_013390	p.Val998Ala	84
USH1G	**NM_173477**	**p.Leu399Alafs*24**	**111**
SSB	NM_003142	p.His373_Glu375del	78

Family DF103

Gene	Refseq	Mutation	Number of reads
MYO7A	**NM_000260**	**p.Glu1812Lys**	**93**
GSTT1	NM_001127180	p.Arg94Cys	36
TMEM52	NM_178545	p.Leu24_Leu26del	7
